# Development of a chitosan/polyethylene oxide/ginger nanocomposite: structural characterization and antibacterial performance

**DOI:** 10.1039/d6ra04293g

**Published:** 2026-08-05

**Authors:** Ghada A. Mostafa, D. M. Ayad, A. A. Menazea

**Affiliations:** a Chemistry Department, Faculty of Science, Mansoura University Mansoura 35516 Egypt; b Spectroscopy Department, Physics Research Institute, National Research Centre Dokki 12622 Giza Egypt aanter7@gmail.com

## Abstract

The development of bio-based antibacterial materials using natural additives is a crucial strategy for reducing reliance on hazardous synthetic chemicals. This study investigated the effect of incorporating ginger nanoparticles (GNPs) at varying concentrations (1, 2, 3, and 4 mL) into a chitosan/polyethylene oxide (PEO) blend to enhance its antibacterial properties. XRD analysis revealed that hydrogen bonding between chitosan and PEO produced a broad peak at 23.60°. The addition of the GNPs intensified this peak and introduced a sharper feature at 19.00°, confirming the successful interaction between the nanoparticles and the polymer matrix. FTIR spectroscopy showed new C–O–C vibrational bands at 1107 cm^−1^ upon blending, with further spectral changes observed after GNP addition, indicating the formation or disappearance of specific functional groups. The incorporation of the GNPs was demonstrated by a new absorption peak at 235 nm, and the semi-crystalline nature of the nanocomposite was confirmed by optical analysis. TEM revealed that the GNPs had diameters of 36.6 ± 13.8 nm, while zeta potential analysis at 25 °C recorded count rates of 106.8, 71.90, and 83.50 kcps for three distinct particle bands, and the mean was −19.33 mV, with a zeta potential deviation of 14.0 mV. The value of the zeta potential indicated the moderate stability of the GNPs. SEM demonstrated that increasing the GNP concentration led to larger, aggregated particle morphologies, with complete coalescence at the highest concentration. The chitosan/PEO blend exhibited higher thermal stability than pure chitosan, while the final polymer nanocomposite showed reduced residue levels at elevated temperatures. Antibacterial testing against Gram-positive (*Enterococcus* and *Staphylococcus aureus*) and Gram-negative (*Escherichia coli* and *Klebsiella*) bacteria demonstrated that the GNP-loaded nanocomposite exhibited enhanced antimicrobial activity compared with the pure polymer blend. For Gram-negative bacteria, the activity was small or negligible. These results position the chitosan/PEO/GNP nanocomposite as a promising bio-based material for antibacterial applications.

## Abstract 2

This study investigates the effect of incorporating ginger nanoparticles (GNPs) with various concentrations into chitosan/polyethylene oxide (PEO) blend to enhance its antibacterial properties.

## Graphical abstract

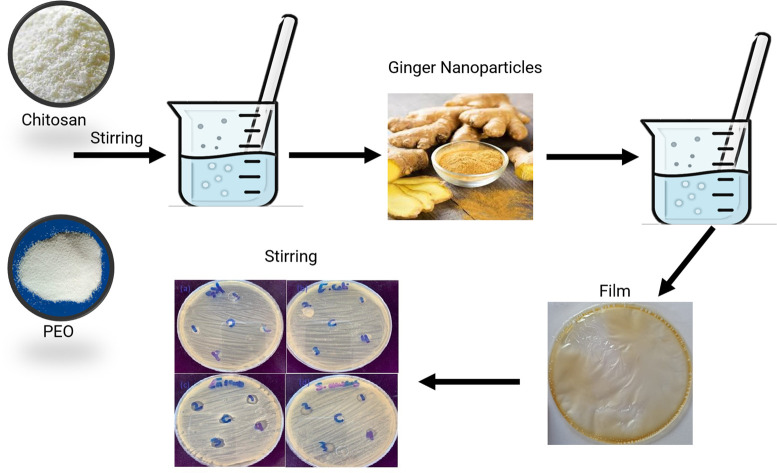

## 1 Introduction

The world is increasingly focusing on harnessing natural resources, such as herbs, to prepare food, medicine, and other necessities. One of them is ginger, which is the focus of this study. Ginger is well-known across the world and has many uses. Marjan Nouri *et al.* reported that ginger has been used for medicinal needs, such as to treat cancer, heart disease and skin conditions, because of its antioxidant and antimicrobial effects.^1^ Ginger has a variety of uses for many illnesses, such as headaches, inflammation and discomfort.^2^ It has many therapeutic properties, such as antiemetic, antithrombotic, anticarcinogenic, antibacterial, antifungal, antidiabetic, and antioxidant effects. It stimulates granulomatous inflammation, which is the main pathological sequelae related to *Schistosoma* infection.^3^ The synthesis process of ginger nanoparticles is less complicated than that of polymer-based nanoparticles.

Chitosan is a derivative of chitin, which is its main source, and it is prepared by a deacetylation process.^4^ Chitin is a polysaccharide found in nature, particularly in the exoskeletons of a variety of organisms such as insects, lobsters, shrimps, and crabs. In shellfish, demineralization and deproteinization are performed using HCL and NaOH, respectively.^5^ Chitosan has the advantages of being biodegradable, less toxic, and biocompatible, and hence, it is used in water treatment, implant surgeries, food packaging, drug delivery, cosmetics and other applications.^6–8^ Because the d-glucosamine residues in the polymeric structure of chitosan can undergo protonation, they enable chitosan to form a polycationic molecule at the physiological pH. Chitosan is soluble in acidic solutions but is insoluble in water, which makes it suitable for mucosal drug delivery applications, such as ocular and intranasal administration, due to its mucoadhesive properties.^9^

Polyethylene oxide (PEO) is a polyether in which the C–O bond is considered one of the least reactive and sensitive bonds. It is considered an important candidate for solid polymer electrolytes (SPEs) because of its semi-crystalline nature and good solubility of alkaline solvents.^10^ Crystals prevent electron transfer; therefore, to improve ionic conductivity, the content of the amorphous phase is increased, while the crystalline phase is minimized. Copolymerization, branching, blending, and nanoparticle incorporation are performed to solve this problem.^11^ When crystals are reduced, mechanical properties are lost. Because of its low toxicity, PEO is considered a good polymer for devices in contact with living organisms.^12^

The blending of chitosan with PEO improves the interference in chitosan chains and changes the physical properties compared to pure chitosan. H. M. Alhusaiki Alghamdi *et al.* studied chitosan and PEO blends with different concentrations of copper oxide for use in optoelectronic devices, resulting in enhanced crystallinity.^13^ FTIR and XRD analyses confirmed the compatibility between the two polymers, as new bands appeared at 709 cm^−1^ and 483 cm^−1^. A. A. Menazea *et al.* studied iron oxide nanoparticles embedded in chitosan/PEO polymer blends by laser ablation and reported a decrease in crystallinity due to nanoparticle interactions, based on XRD analysis.^14^ The polymer nanocomposite improved the electrical features, promoting the electrical applications of nanocomposites.

Sawsan Ali Al-Hilifi *et al.* used ginger essential oil with chitosan for the fabrication of bio-based food packaging materials.^15^ To avoid contamination, the use of natural antimicrobial materials is in demand. Blending chitosan with ginger can make edible films suitable for food packaging and enhance chitosan's antimicrobial effects. Augustine Amalraj focused their attention on wound dressing and food packaging using ginger and black pepper essential oil with polyvinyl alcohol, chitosan and gum Arabic.^16^ They reported that after the incorporation of essential oils, the films formed were resistant to breakage, flexible and stable under heat. According to antibacterial tests, there was an inhibition in bacterial growth (*Bacillus cereus*, *Staphylococcus aureus*, *Escherichia coli* and *Salmonella typhimurium*). Mabrouka Ghabbari and Awatef Elwej (2026) combined chitosan with gelatin and *Zingiber officinale* herb (CH + GE + ZO) to prepare a wound-healing dressing for diabetic patients. They found that the prepared dressing improved the healing process compared to the control, and after 13 days, total recovery was observed. It ended with progressive re-epithelialization, a structured organization of the layers of the epidermis, and a partial restoration of the cutaneous appendages.^17^ Xiaohan Wang and Zhaohui Xue (2024) combined chitosan with ginger essential oil (GEO) nanoemulsions to prepare films for blueberry preservation. They found that there was an improvement in the mechanical, thermal and barrier properties (water vapor permeability improved from 1.52 × 10^−9^ to 6.54 × 10^−10^ g m Pa^−1^ s^−1^ m^2^). Antibacterial and antioxidant activities were observed with the release of GEO. The films formed had significant potential in biodegradable material development for food preservation.^18^

In this study, we study the effect of the addition of the GNPs to a chitosan/PEO blend to improve the chemical and antibacterial properties of the chitosan/PEO blend. We have used XRD, FTIR spectroscopy, SEM, TEM, and antibacterial tests to study the effect of the GNPs on the chitosan/PEO blend.

## 2 Materials and method

### 2.1. Materials

Chitosan [(C_6_H_11_NO_4_)_*n*_, 85%, deacetylated, average molecular weight = 941.58 g mol^−1^, powder form] was purchased from Alfa Aesar, 26 Parkridge Road, Ward Hill. PEO (average molecular weight = 40 000 g mol^−1^, powder form) was purchased from LANXESS, Germany.

### 2.2. Methods

#### 2.2.1. Ginger nanoparticle preparation

The preparation of the GNPs included multiple steps. First, a high-quality ginger extract was obtained by solvent extraction, which was dried to dissolve the lipophilic bioactive compounds. Then, the powdered ginger was refluxed with ethanol at 60 °C for 1 hour. The solution formed underwent filtration and concentration using a rotary evaporator to obtain a viscous ginger oleoresin. The nanoprecipitation step involved dissolving the concentrated oleoresin in a water-miscible solvent (ethanol) to get an organic phase and an aqueous phase consisting of water, containing a surfactant, such as Tween 80, to stabilize the nascent nanoparticles. The nanoparticles were formed by injecting the organic phase rapidly into the aqueous phase (the antisolvent) and stirring vigorously, resulting in sudden supersaturation and the precipitation of the ginger bioactives as nanoscale particles. The crude nanosuspension was subjected immediately to probe ultrasonication (at 20 kHz for 10 min in an ice bath to prevent overheating), which utilized high-intensity sound waves to generate intense shear forces, which break down large aggregates, reducing the size of particles to achieve a more homogeneous distribution. Finally, using reduced pressure, the organic solvent was removed from the nanosuspension, the resulting colloidal dispersion of the GNP centrifugate was purified, and excess surfactant was removed by washing.

#### 2.2.2. Chitosan/PEO blend preparation

Chitosan preparation: 2 g of chitosan was mixed with 400 mL of distilled water and 1 mL of acetic acid and stirred for 3 days till complete dissolution.

PEO preparation: 2 g of PEO was added to 200 mL of distilled water and left on a stirrer overnight.

Polymer blend: 300 mL of the CS polymer was added to 150 mL of the PEO polymer and left to stir for 1 hour. The rest of the pure polymers was poured into Petri dishes. After blending, the blend was separated into 5 parts; each part contained 90 mL of the blend. The first 90 mL was poured into a Petri dish, and the rest was used to prepare the chitosan/PEO blends and nanocomposites.

#### 2.2.3. Chitosan/PEO/ginger nanocomposite preparation

The synthesized GNPs (at a concentration of 10 mg mL^−1^) were added to the beakers with the polymer blend, with 1 mL, 2 mL, 3 mL or 4 mL of the GNPs in every 90 mL of the blend. Then, it was poured into Petri dishes. All Petri dishes were incubated in an oven at 50 °C for 3–5 days and then left in fresh air for 3 days. The films were ready to be characterized. The names of the samples were abbreviated as follows: Blend: chitosan/PEO, BG1: chitosan/PEO/1 mL GNPs, BG2: chitosan/PEO/2 mL GNPs, BG3: chitosan/PEO/3 mL GNPs, BG4: chitosan/PEO/4 mL GNPs.

### 2.3. Characterization

X-ray diffraction has been performed *via* a PANalytical X-Pert PRO X-ray diffractometer, employing copper Kα radiation (45 kV, *λ* = 1.5403 Å). The Bragg angles have been measured across a diffraction range of 5–70°. Fourier transform infrared absorption spectra have been acquired using a Bruker FT-IR spectrophotometer (Invenio S, Germany) with a resolution of 4 cm^−1^ within the spectral domain of 4000–400 cm^−1^. The UV/Vis absorption spectra of polymer films were recorded using spectrophotometer Shimadzu 1601, Japan, in the wavelength range 200–1000 nm. The morphology of the synthesized films has been investigated *via* scanning electron microscopy (SEM) using a JEOL JSM-6510 in the high-vacuum mode with a 30 kV accelerating voltage. Transmission electron microscopy (TEM) has been investigated on a JEOL-2100 EX instrument (Tokyo, Japan), operating at an accelerating voltage of 120 kV. TGA was executed using a Shimadzu TGA-45H under a nitrogen atmosphere, with a linear temperature ramp from 30 to 600 °C at a rate of 5 °C per minute.

### 2.4. Antimicrobial test using the disc diffusion method

**Fig. 1:**
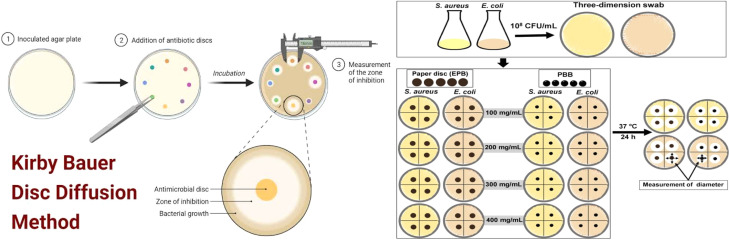
Kirby–Bauer disc diffusion method for studying the antibacterial effect.

Antimicrobial tests of the prepared chitosan/PEO/ginger nanocomposite films were performed *via* the disc diffusion method, as shown in Fig. 1. Two Gram-positive bacteria (*Enterococcus* and *Staphylococcus aureus*) and two Gram-negative bacteria (*Escherichia coli* and *Klebsiella*) were used. The disc diffusion method is a qualitative method for studying the effect on bacterial growth. The LB agar medium was liquefied by heating till it melted and then poured in sterilized Petri dishes; then, the bacteria were spread in plates. Film discs (about 0.5 × 0.5 cm), sterilized with UV light, were put in agar plates that had cultured bacterial colonies, which were incubated overnight at 37 °C to see the effect. This method is called the disc diffusion method.^19^ Then, the inhibition zones formed were measured, and the activity index was calculated using the following equation.^20^Activity index = (zone of inhibition by the test compound (diameter)/zone of inhibition by the standard (diameter)) × 100

## 3 Results and discussion

### 3.1. Characterization of ginger nanoparticles

**Fig. 2:**
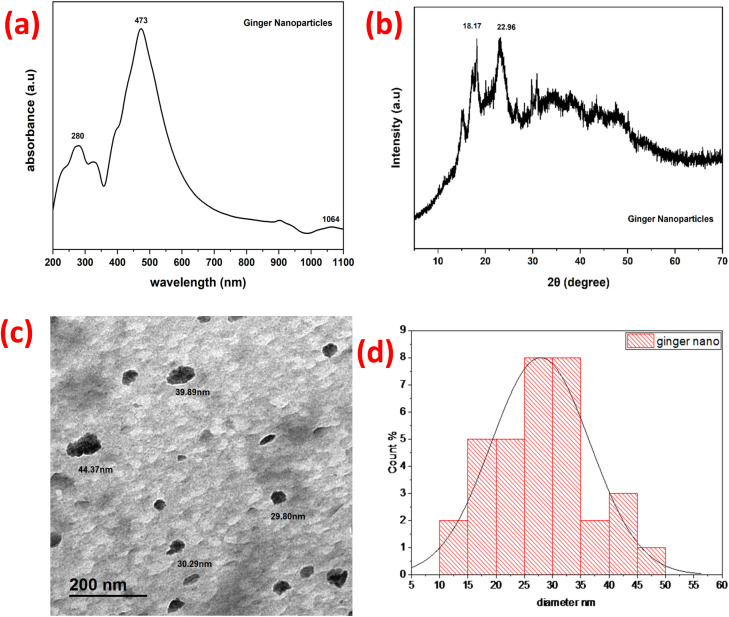
(a) UV-Vis spectrum, (b) XRD pattern, (c) TEM image, and (d) histogram of the size distribution of the GNPs.

The characterization of the GNPs is shown in Fig. 2. Fig. 2a shows its UV-Vis spectrum, which shows three peaks; the broad and highest peak indicates the maximum absorption at 473 nm. XRD analysis, as shown in Fig. 2b, shows two bands of GNPs at 18.17° and 22.96°, with the most obvious and sharp band at 22.96°. Fig. 2c shows that the average size of the GNPs is 36.6 ± 13.8 nm. The TEM images show that the GNPs are spherical have a wide size distribution and are slightly agglomerated. According to the histogram in Fig. 2d, there is a variation in the particle size. About 8% of the particles have a diameter of 25 to 35 nm, which confirms the successful isolation of the GNPs, making them stable for use. Zeta potential analysis was used to measure the surface charge. For the GNPs, it is found to be −19.33 mV. According to the calculated mean of three measurements, the zeta potential remained stable during storage. When the value of the zeta potential is higher than 25 mV or lower than −25 mV, it means that the material has a higher degree of stability. So, the zeta potential value for the GNPs obtained in this study indicates that they have moderate stability, making their aggregation possible, as illustrated in the TEM image.

### 3.2. Characterization of the chitosan/PEO/ginger nanocomposite films

#### 3.2.1. FTIR

**Fig. 3:**
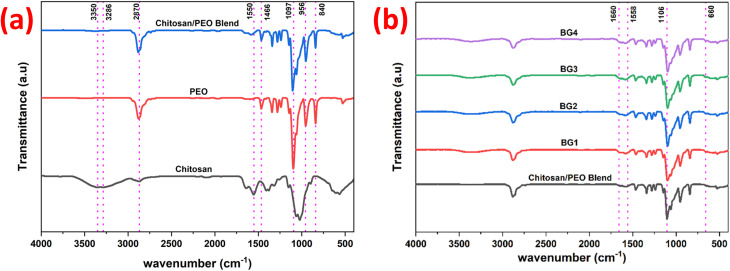
FTIR spectra of (a) chitosan, PEO, and the chitosan/PEO blend and (b) chitosan/PEO blend and chitosan/PEO blends filled with various contents of the GNPs.

The FTIR spectra of chitosan, polyethylene oxide and the blend are shown in Fig. 3a. The chitosan spectrum shows the following bands: the vibrational bands at 3350 cm^−1^ and 3286 cm^−1^ indicate N–H and O–H, respectively, in addition to intermolecular hydrogen bonding,^21^ the stretching band at 2870 cm^−1^ indicates C–H asymmetric vibrations, and the bending N–H vibration (amide 11) appears at 1550 cm^−1^. The stretching band at 1376 cm^−1^ indicates symmetrical CH_3_, bridged C–O–C vibrations appear at 1154 cm^−1^, and the vibrational band at 1058 cm^−1^ indicates -C-O stretching.^22^ The PEO spectrum shows a band at 2870 cm^−1^, corresponding to saturated asymmetric C–H in methylene groups, and the bands at 1466 cm^−1^ and 1339 cm^−1^ indicate CH_2_ in both scissoring and asymmetric bending modes, respectively. The C–O–C stretching vibration band is obtained at 1097 cm^−1^, the band at 1058 cm^−1^ ascribed to the C–C bond, and the bands at 956 cm^−1^ and 840 cm^−1^ are attributed to CH_2_ in the rocking mode and C–O, respectively.^23^Fig. 3a shows a deviation from the chitosan spectrum, except for the band at 1154 cm^−1^, which shifts slightly to the right. A deviation from the PEO spectrum appears for the band at 1107 cm^−1^, which corresponds to C–O–C, and the rest of the bands are the same.

The changes in the polymers after adding the nanoparticles, compared to the blend, appear as new bands in the spectrum, as shown in Fig. 3b. The weak band at 1660 cm^−1^, ascribed to C=C alkene, appears in all spectra except BG1; the stretching band at 1588 cm^−1^, ascribed to aromatic C=C, appears in BG1 and BG3.^24^ The strong stretching band at 1106 cm^−1^, corresponding to C–O–C, appears in BG1 only,^25^ and the stretching band at 1099 cm^−1^, ascribed to C–OH, disappears in BG1. The band at 990 cm^−1^, corresponding to the wagging C–H of alkenes, appears in BG1 and BG3. The band at 660 cm^−1^, corresponding to C–H bending, appears in all samples except BG2.

#### 3.2.2. XRD analysis

**Fig. 4:**
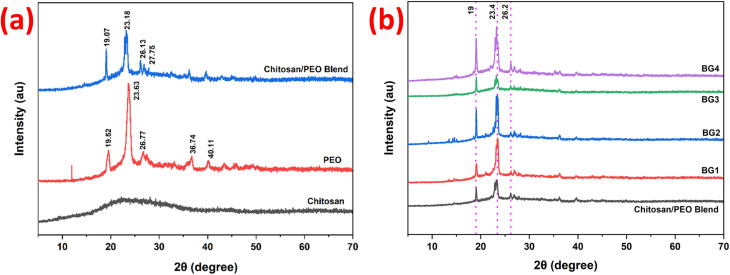
XRD patterns of (a) chitosan, PEO, and the chitosan/PEO blend and (b) the chitosan/PEO blend and chitosan/PEO blends filled with various contents of the GNPs.

As shown in Fig. 4a, chitosan shows a broad hump at 2*θ* = 15–30°, which corresponds to the amorphous nature of chitosan. The spectrum of PEO confirms the semi-crystalline nature of PEO and shows high-intensity peaks at 2*θ* = 19.52°, 23.6°, and 26.77°,^26,27^ related to (112), (120), and (222), respectively, along with low-intensity peaks at 2*θ* = 36.74° and 40.11°. The chitosan/PEO blend shows two sharp peaks at 2*θ* = 19.52° and 23.3, originating from the semi-crystalline nature of PEO, and the 23.6° peak seems to be broader than that in PEO, resulting from the hydrogen bonding interaction between chitosan and PEO.^28^

As shown in Fig. 4b, the addition of the GNPs to the polymer blend causes the intensity of the peak at 2*θ* = 19.0° to increase more than that of the blend, and it becomes sharper than the blend with increasing concentration of the GNPs. Archana *et al.*, in 2022, studied a ginger nanopowder and observed that 2*θ* values for ginger were in the range of 10° to 50° at a 2°/min scan rate.^29^ This figure shows that with an increase in the GNPs, the intensities of the peaks increase compared to the polymer blend. The peak at 2*θ* = 21.2° at high concentrations of GNPs appears more intense, and in BG3, the peak appears smaller. The Debye–Scherrer formula was used to calculate the average crystallite size of CS/PEO with the GNPs using the diffraction angle (2*θ*) and full width at half-maximum (FWHM) of the diffraction peaks, as follows: *D* = *K* × *λ*/*β* cos *θ*, where *K* is a constant (0.91), *λ* is the X-ray wavelength (0.154 nm), *β* is the FWHM and *θ* is the diffraction angle.^30,31^

According to this equation, the crystal size of the GNPs decreases in the chitosan/PEO blend, and at 2*θ* = 22.96°, it is 12.67 nm.^29^

#### 3.2.3. Optical analysis

**Fig. 5:**
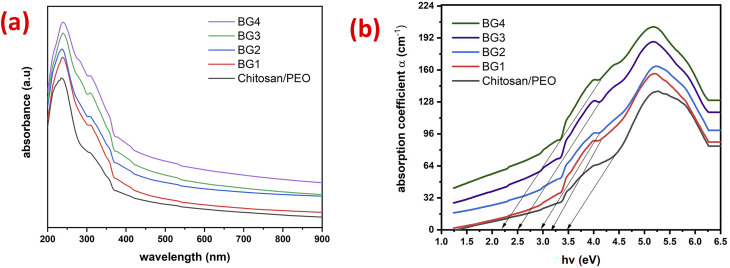
(a) UV-visible absorbance spectra and (b) the relation between the absorption coefficient (*α*) and the photon energy (*hν*) of chitosan/PEO blends filled with various contents of the GNPs.

As shown in Fig. 5a, the UV spectra of the chitosan/PEO blend and the chitosan/PEO blend filled with various contents of the GNPs were recorded in the wavelength range from 200 to 1000 nm. GNPs shifted the absorption wavelength of chitosan/PEO. Absorption for the blend were observed at 235 nm, which were used to confirm the semi-crystalline behavior of nanocomposites.^26^ Energy is absorbed by materials in the UV spectrum due to electron transmission from the ground to the excited state, and the absorption band observed at 308 nm was related to π–π* bonding. The Beer–Lambert equation, *α* = (2.303 × *A*)/*d*, where *A* is the absorbance and *d* is the film thickness, was used to calculate absorption coefficients to find the energy gap.^32,33^

**Fig. 6:**
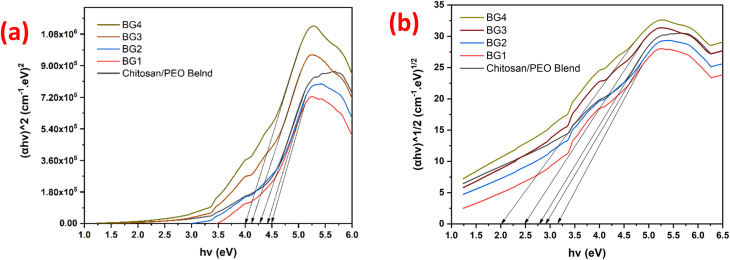
(a) Plot of (*αhv*)^2^ against the photon energy (*hv*) and (b) plot of (*αhv*)^1/2^ against the photon energy (*hv*) for the chitosan/PEO blends filled with various contents of the GNPs.

The values of absorption coefficients decreased with increasing GNP content in the chitosan/PEO blend, as shown in Fig. 5b. The energy gap is known as the energy difference between the lowest minimum of the conduction band and the highest maximum of the valence band. If the conduction band minimum and the valence band maximum occur at the same position, it is called a direct gap; if they occur at different positions, it is called an indirect gap. As shown in Table 1, the values of direct and indirect optical band gaps decreased with an increase in the GNP concentration, indicating that the GNPs would increase the number of energy states between the chitosan/PEO valence and conduction bands, so that the electronic structure of the chitosan/PEO would be modified. As shown in Table 1, the direct energy gap increased from 2.7 to 3.3 eV compared with the pure blend and then decreased as the GNPs increased, until it reached 2.0 eV for BG4. The same is observed for the indirect energy gap, as it increased from 4.39 to 4.53 eV when 1 mL of the GNPs was added and then decreased as the GNP concentration increased, till it reached a band gap of 4.12 eV for BG4. Indeed, this change in optical band gap can be attributed to the localized electronic states introduced into the band gap of the chitosan/PEO blend by the GNPs, which act as trapping and recombination centers. These localized density states are related to the concentration defects, leading to a decrease in the optical band gap energy. Also, these decreases provide evidence of the increase in the degree of disorder in chitosan/PEO samples. Direct and indirect energy gaps were obtained by plotting (*αhν*)^2^ and (*αhν*)^1/2^ against *hν*, respectively, as shown in Fig. 6a and b.

**Table 1:** Values of the direct and indirect energy gaps and absorption coefficients of the pure blend (chitosan/PEO) and the blends doped with the GNPs

Sample	Ginger nanoparticles	Direct energy gap (eV)	Indirect energy gap (eV)	Absorption coefficient
Chitosan/PEO blend	0	2.7	4.39	3.5
BG1	1 mL	3.3	4.53	3.26
BG2	2 mL	2.8	4.45	2.93
BG3	3 mL	2.5	4.22	2.5
BG4	4 mL	2.0	4.12	2.23

#### 3.2.4. SEM analysis

**Fig. 7:**
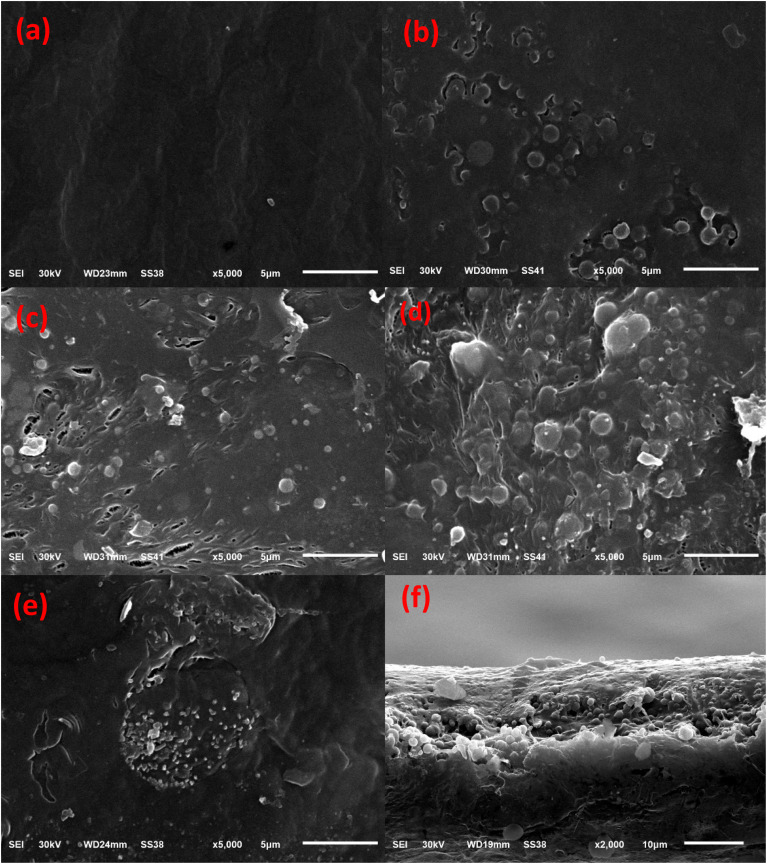
SEM photographs of the (a) pure chitosan/PEO matrix, chitosan/PEO matrix filled with different concentrations of natural GNPs: (b) 1 mL of GNP, (c) 2 mL of GNP, (d) 3 mL of GNP, and (e) 4 mL of GNP, and (f) cross-section of the chitosan/PEO matrix doped with 4 mL of the GNP sample.

SEM analysis was used to describe the dispersion of polymer particles in the polymer blend and observe how the nanoparticles mixed with the polymer blend. The image of the chitosan/PEO matrix shows a smooth and uniform morphology, indicating the compatibility of the two polymers, as shown in Fig. 7a. SEM was used to study the dispersion of the GNPs in the chitosan/PEO blend. For the blends containing 1 mL of GNPs, the image shows circular-shaped particles that aggregate with each other. With GNP concentration increases the aggregation and particles increase and become bigger in size as shown in Fig. 7d. Fig. 7e shows that the particles agglomerate into capsule-like structures and shrink into distinct domains which indicates the interaction between GNPs and chitosan/PEO blend and illustrates that high concentration of GNPs improve the blending and make the sample almost homogeneous. The cross-sectional image of BG4 in Fig. 7f shows a uniform and compact morphology with an interface between the chitosan/PEO layer and GNP-loaded layer, entrapped with aggregates of the GNPs, which may be due to inhomogeneous dispersion or density differentiation.

#### 3.2.5. Thermal analysis

**Fig. 8:**
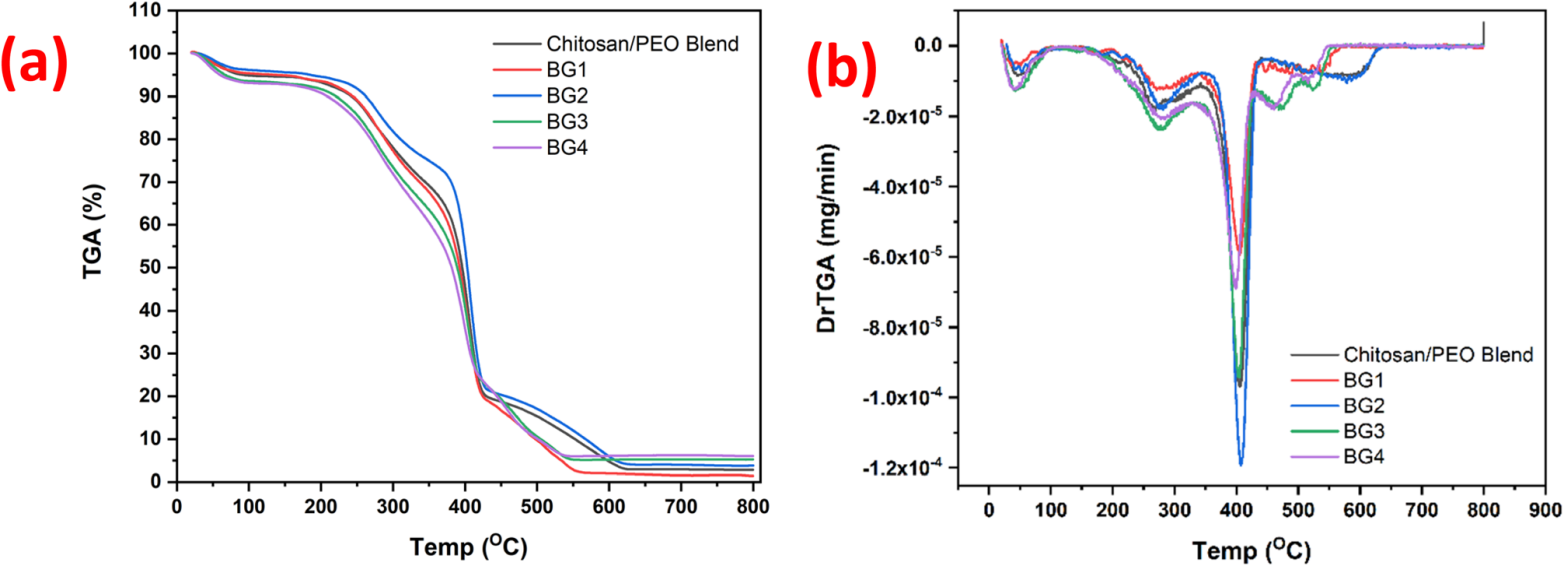
(a) Relation between TGA and temperature for the chitosan/PEO blend and chitosan/PEO blends filled with various contents of the GNPs. (b) Relation between DrTGA and temperature for the chitosan/PEO blend and chitosan/PEO blends filled with various contents of the GNPs.

Thermogravimetric analyses (TGA and DTGA) were used to investigate the thermal stability of CS, PEO and the GNPs. TGA curves show that CS degrades in two stages: the first stage is the loss of water that was not removed during drying, and the second stage is the combustion of the chitosan chain. PEO degrades in one step at about 350 °C, indicating the thermal stability of PEO. The blend starts to degrade at 350 °C, corresponding to degradation of PEO, and 440 °C, corresponding to the degradation of CS.^34^ The addition of the GNPs to the polymer blend increases the temperature of decomposition. BG2 shows a degradation profile similar to that of the blend, indicating comparable thermal stability, which makes it resistant to heat decomposition. Furthermore, the other nanocomposites showed increased stability, with degradation initiating at about 490 °C. The thermal stability increased with increasing GNP concentration, resulting in higher decomposition temperatures. DTGA was used to confirm the TGA data. PEO shows a sharp degradation peak around 400 °C; meanwhile, CS has a small, narrow peak, and the blending shifts the peaks to higher temperatures, as shown in Fig. 8. BG2 shows the sharpest peaks compared to the other concentrations, which means that it has the highest stability. The data indicate that blending CS and PEO, followed by the addition of different concentrations of the GNPs, can improve thermal properties, especially with the addition of 2 mL of GNPs.

#### 3.2.6. Antibacterial activity

**Fig. 9:**
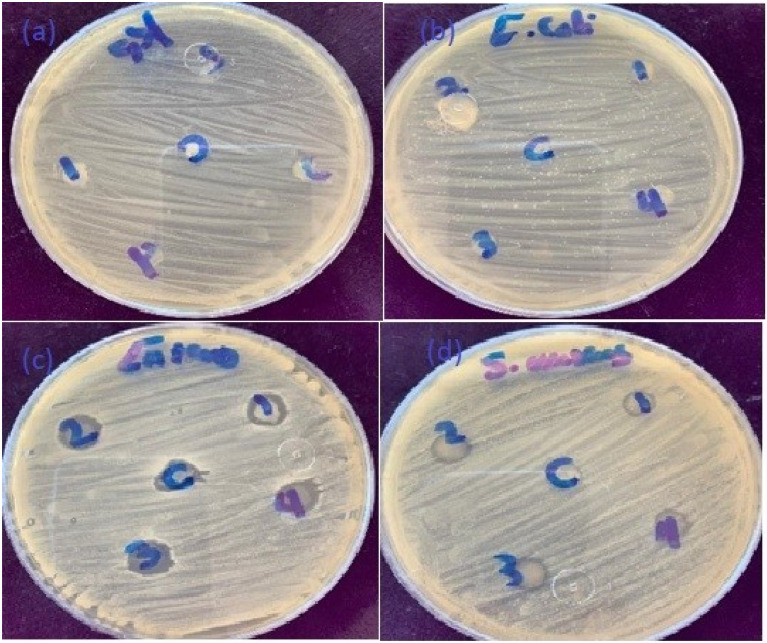
Disc diffusion antibacterial test on (a and b) Gram-negative bacteria and (c and d) Gram-positive bacteria for chitosan, PEO, and the chitosan/PEO blends; the letter c represents the chitosan/PEO blend, and numbers 1 to 4 represent the chitosan/PEO blend filled with various contents of the GNPs.

The antibacterial test was done to ensure the efficiency of the tested nanocomposite before being used in medical or other applications. The zone of inhibition formed by the pure polymer blend and the blend doped with four concentrations of the GNPs against *Enterococcus* and *Staphylococcus aureus* (Gram-positive) and *Escherichia coli* and *Klebsiella* (Gram-negative) is given in Table 2. As seen in Fig. 9, the polymer blend (chitosan/PEO) and polymer nanocomposite, chitosan/PEO/GNPs (1, 2, 3, and 4 mL), had a small effect on Gram-negative bacteria. On the other hand, there was a great effect against Gram-positive bacteria. As shown in Table 2, in the case of *S. aureus*, the inhibition zone was 9 ± 1.3 mm, and the effect of doping GNPs increased with increasing concentration until the inhibition zone reached 12 ± 1.3 mm. The chitosan/PEO blend had a negligible effect initially, and the effect became more pronounced after the addition of increasing concentrations of the GNPs. In *Enterococcus*, the zone of inhibition for the pure polymer was 11 ± 1.3 mm and was larger than that of *S. aureus*, and it increased after the GNPs were doped in the polymer blend at different concentrations, finally reaching a value of 13 ± 1.3 mm. It was observed that adding GNPs raised the efficiency of the chitosan/PEO polymer blend against Gram-positive bacteria, as zingiberene and α-curcumene, which are the main components of ginger, work on cell membranes by damaging them.^35^ The enhanced antibacterial efficacy against Gram-positive bacteria can be attributed to their lack of an outer lipopolysaccharides membrane, which is present in Gram-negative bacteria.

**Table 2:** Values of the inhibition zone formed by the chitosan/PEO blend filled with various contents of the GNPs against Gram-positive and Gram-negative bacteria

Sample type	Microorganism			
	Diameter of the inhibition zone (mm)			
	Gram-positive		Gram-negative	
	*Enterococcus*	*Staphylococcus aureus*	*Escherichia coli*	*Klebsiella*
Chitosan/PEO	11 ± 1.3	9 ± 1.3	2 ± 0.9	1.5 ± 0.8
BG1	10 ± 1.3	8 ± 1.3	3 ± 0.1	2 ± 0.08
BG2	9 ± 1.3	10 ± 1.3	2 ± 0.09	3 ± 0.1
BG3	12 ± 1.3	11 ± 1.3	2 ± 0.15	2 ± 0.1
BG4	13 ± 1.3	12 ± 1.3	3 ± 0.07	3 ± 0.2

## 4 Conclusion

This study aims to study the optical, morphological and antibacterial properties of chitosan by blending with polyethylene oxide (PEO). Blending a natural polymer (chitosan) with a synthetic polymer (PEO) improves its properties. Using natural herbs is a need nowadays owing to their natural benefits in many places. Ginger is considered an antimicrobial and antitoxic agent, and the GNPs have the ability to be mixed with polymer blends. XRD and FTIR analyses show that the addition of the GNPs results in the appearance of new bands due to the presence of new groups, such as the formation of new C–O–C and alkene and aromatic C=C bonds, or the absence of functional groups, indicating the blending of the polymer blend with the nanoparticle. When the GNP concentration increases, the energy gap increases, as shown in the optical test. The decrease in band gap is related to the disorder degree in the chitosan/PEO samples. Morphological analysis shows that the diameter of the GNPs ranges from 29 to 44 nm, and with an increase of the GNP concentration, the polymer nanocomposite's particle size increases, as illustrated by SEM analysis. We have performed zeta potential analysis to confirm the TEM results of the GNPs. It shows that the mean average size is 36.6 ± 13.8 nm. The blending improves the properties of the polymer blend, as determined by applying the polymer nanocomposites to Gram-positive and -negative bacteria and studying the effect on each one. The nanocomposite shows activity toward Gram-positive bacteria, and the inhibition zones formed range from 9 ± 1.3 to 12 ± 1.3 mm for *S. aureus* and 11 ± 1.3 to 13 ± 1.3 mm for *Enterococcus.* It is observed that adding the GNPs raises the efficiency of the chitosan/PEO polymer blend against Gram-positive bacteria, which could suggest the potential of the prepared samples for wound-healing applications.

## Author contributions

Ghada A. Mostafa: methodology, investigation, writing – original draft, writing – review and editing. D. M. Ayad: supervision, investigation, writing – original draft, writing – review and editing. A. A. Menazea: methodology, investigation, writing – original draft, writing – review and editing.

## Conflicts of interest

The authors declare that they have no conflicts of interest.

## Data Availability

The data will be available on request.
